# Depressive Symptoms Attenuate the Cognitive Benefits of Slow Wave Sleep in Older Black Americans

**DOI:** 10.1093/geroni/igaf122.4096

**Published:** 2025-12-31

**Authors:** Stirling Crawford, Osswaah Fariduddin, Maria Clark, Elizabeth Luth, Darlingtina Esiaka

**Affiliations:** University of Kentucky, Frankfort, Kentucky, United States; University of Kentucky, Lexington, Kentucky, United States; University of Kentucky, Lexington, Kentucky, United States; Rutgers University, New Brunswick, New Jersey, United States; University of Kentucky - College of Medicine, Lexington, Kentucky, United States

## Abstract

**Background:**

Slow wave sleep (SWS), critical for memory consolidation, has been linked to better visual-spatial working memory (VSWM)—a sensitive early marker of cognitive decline. Depressive symptoms have been shown to modulate memory performance, but their interactive effects with SWS remain underexplored in Black American populations disproportionately impacted by sleep disturbances. This study investigated the interaction between objectively measured SWS and depressive symptoms on VSWM among older Black Americans.

**Methods:**

As part of a larger observational study examining longitudinal benefits of sleep in older Black Americans, participants (N = 59; mean age 52.76) completed three consecutive nights of at-home sleep monitoring using the Sleep Profiler, a validated wearable device. VSWM performance was assessed using standardized memory tasks, while depression was assessed using the Geriatric Depression Scale. Moderation analysis was used to examine whether depression moderated the relationship between SWS and VSWM, while controlling for age, gender and marital status.

**Results:**

SWS (B = 0.665, p = 0.006) and depressive symptoms (B = 2.177, p = 0.008) had significant independent effects on VSWM. The moderation analysis revealed a significant interaction (B=-0.1106, p = 0.007) between SWS and depressive symptoms. Further examination of the conditional effects revealed a positive association between SWS and VSWM at low depressive symptom levels (t = 2.8847, p = 0.0057) and a negative association at higher depressive symptom levels (t=-2.7760, p = 0.0076).

**Conclusions:**

These findings suggest that while SWS supports VSWM at low levels of depressive symptoms, the benefit diminishes under high depressive symptoms. Thus, emphasizing the need to investigate sleep–mood interactions when assessing the benefits of sleep among aging Black Americans.

